# Characteristics and Health of Turkey Husbandry in Ouaké, North-Benin

**DOI:** 10.5402/2011/723091

**Published:** 2010-12-14

**Authors:** E. Y. Attakpa, L. G. Aplogan, A. Y. J. Akossou, R. H. Bosma

**Affiliations:** ^1^Faculté d'Agronomie, Université de Parakou, BP 123 Parakou, Benin; ^2^Laboratoire de Diagnostic Vétérinaire et de Sérosurveillance, BP 23 Parakou, Benin; ^3^Animal Sciences Group, Wageningen University, P.O. Box 338 6700AH, The Netherlands

## Abstract

Sanitary constraints of raising turkey in north-west Benin were studied by using a survey and Haemagglutination Inhibition Test (HIT) to detect antibodies of Newcastle Disease (ND) and Avian Influenza (AI). We tested 85 serums from 7- to 24-month-old turkeys raised in 19 farms. ND prevalence rate was 54% but reactions on four sub-types of AI were negative. Mortality rates varied from 55 to 100% for 0–30 day-old flocks; 30% for 1- to 4-month-old; and 15% for older turkeys. Next to ND, probable causes of mortality are Fowl pox, Gumboro disease, scabies, coccidiosis, histomonosis, capillariosis and colibacillosis. Only one farmer who fed and vaccinated the poults, and provided clean housing for them got a lower mortality rate of 11% in turkeys less than 4-month-old. The question remains why most farmers do not apply these simple practices: are they unaware or are the technologies not profitable?

## 1. Introduction

Poultry keeping is a widespread traditional activity in Africa. It provides protein supplement for households and gives them a stock for urgent needs [1]. Poultry plays a role in many traditional ceremonies and festivals, and scavenging poultry reduces the population of vermin [2]. Increasing productivity of poultry husbandry may help reduce poverty and improve household's food safety [3, 4]. A participatory diagnosis carried out with the breeders in the commune of Ouaké identified mortality as the major limiting factor in turkey husbandry.

In several African countries, the village poultry production level is suboptimal mainly due to predation and contagious diseases [5]. In Benin, which has approximately 14 million poultries [6], Newcastle disease (ND) was clinically confirmed in chicken [7]; its prévalence was 67% for local chickens in Atacora and Donga [8]. ND was recognized as the major constraint generating 70% of officially recorded poultry mortalities [6]. Serum antibodies against the avian influenza virus (AI) were detected in chicken even in the absence of any vaccination [9]. 

Indeed, in their extensive husbandry system, scavenging turkeys are exposed to various infectious agents through contact with other poultry species and birds. Farmers in Ouaké, known for their turkey husbandry, have noticed increasing mortality and decreasing flock sizes in recent years. Information on the immunological profiles of these two viral pathologies is missing, however. The hypothesis of the present study is that ND and AI are the principal constraints of turkey production in Ouaké.

## 2. Material and Methods

The commune of Ouaké is located between 9° and 10° north and 1° and 2° east and covers a surface of 1500 km^2^. Ouaké neighbours Togo and belongs at present to the department of Donga but formerly it was the southern point of Atacora. Ouaké has a humid Sudanian climate with two seasons and an average temperature of 27°C. The rainy season falls from May to October. 

We selected 19 husbandries from among voluntary turkey breeders raising more than 3 animals in 7 villages. We surveyed husbandry conditions and clinical descriptions of diseases and mortalities, and collected blood samples of turkeys.

We took serum from 120 turkeys aged between 7 and 24 months. Blood was collected with a syringe and a sterile needle from the wing vein of each turkey and stored in a sterile tube “vacutainer”. After coagulation of the blood at ambient temperature, the serum was transferred in eppendorf tubes at −20°C at the laboratory until testing. 

Considering herd representation, we used a weighted random sample of 85 serums drawn from the 120 turkeys. These serums were analyzed by using the HIT following procedures of the reference laboratory for ND and AI (Instituto Zooprofilacttico Sperimentale delle Venezie, Legnano Padova, Italy). The HIT reaction is based on the property of specific bacteria and viruses to bind red blood cells of poultry due to the presence of agglutinins in the cells. The HIT was carried out in three stages:

- washing of the red globules with PBS, 3 × 5′ in a centrifuge at 6,000 rpm,
- titration of antigens (NDV, H7N3, H9N2, H5N1, and H5N2) of OIE/FAO Laboratory for AI and ND to be used in HIT,
- HIT in NUNC plates of 96 “V”-shaped wells.

In each plate, five serums were analyzed in the first five wells of the first column, the positive and negative serums were placed in wells 6 and 7, and the last well (H1) sheltered the control. The HIT was carried out in the veterinary laboratory (LADISERO) of Parakou. 

The statistical analysis of the data was carried out with Minitab v14. After graphical analysis and calculating proportions, we used the Fisher test for their comparison.

## 3. Results and Discussion

### 3.1. Turkey Husbandry Conditions

Turkey in Ouaké was integrated in traditional poultry keeping. Households keep various species together: chickens, guinea fowls, turkeys, and ducks. In day time, the turkeys are made to scavenge, but at night time, in all visited households, they were housed either in a traditional hen house (90%), or on perches (6%), or in other shelters (4%). 

Recorded losses were caused by various predators: birds (during day time), carnivores, snakes and dogs. Moreover, the poultry were exposed to loss, theft, and disease by wild birds carrying infectious agents. 

Poultry keepers feed their turkeys without considering quantitative and qualitative standards. They give them crushed cereals, and sometimes termites, maggots or kitchen left overs, in addition to what the turkeys have already scavenged during the day. The adult turkeys are given only some wrists of cereal grains on the ground. The mangers and drinking troughs used in the majority of the husbandries are empty cans, broken plates or broken pottery.

In the traditional hen houses, the standards of ventilation, hygiene and density are seldom respected. The average density in Ouaké is 5–8 adults m^−2^, while the standard is 3–4 adults m^−2^. The sanitary status of the housing varies between the households. Sick birds are sold, slaughtered, or treated with inadequate pharmaceutical products or by medicinal plants whose therapeutic virtues are known to some breeders only. Only turkey breeders who have received training in poultry farming try to improve the housing, ensure hygiene in these housings, and follow a vaccination schedule.

### 3.2. Sanitary Status of the Turkey Husbandries

**Figure 1:**
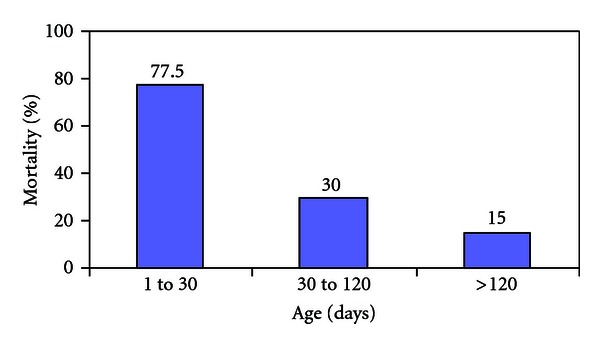
Distribution of the death rates according to three age classes.

The mortality rates are different for three age classes (Figure 1). The death rate among 0-to 30-day-old turkey poults varied between 55 and 100%, and 30% for the 30- to 120- day-old turkey poults. This death rate fell to 15% among 4-month and older turkeys. Death rates of turkeys varied also between farmers and villages, also because some villages had one farmer only. One farmer having attended a training of a poultry project obtained a better result: 11% mortality among 0–4.5-month-old turkey poults. Mortality is higher in the *harmatan* season (low night temperatures, winds carrying dust, when green vegetation and insects are rare).

A preliminary inventory revealed that fowl pox, Newcastle disease, Gumboro disease, coccidiosis, histomonosis (*Histomonas meleagridis*), capillariosis (*Capillariasis philippinensis*), scabies (*Sarcoptes scabiei*), colibacillosis (*Escherichia coli*) prevail in the turkey husbandries of Ouaké. Part of these diseases is related to the husbandry practices: the state of the housing (if existing), the scavenging, the absence of mangers and drinking troughs, insufficient feeds, absence of specific vaccinations and antibiotics.

### 3.3. Newcastle Disease

The average rate of prevalence of specific antibodies of the ND virus was 54% (Table 1). The prevalence rates vary between villages from 40 to 80% with titers of the antibody going from 1 : 16 to 1 : 256. The titers higher than 1 : 16 are regarded as negative for turkeys, species other than chickens. The antigen titer used for ND was 1 : 128. The ND prevalence in gobblers was significantly higher than that in hens: 53.4% and 36.4%, respectively (*P* = .014). Gobblers are more often positive than hens of 17 month and older (Table 2). In view of the small sample size (gobblers 8 on 15 and hens 6 on 16) this difference needs confirmation.

### 3.4. Avian Influenza

The serums were negative to viral sub-types H7N3, H9N2, H5N2, and H5N1 of AI virus (Table 3). This indicates the absence of specific antibodies against these various sub-types of the AI virus in the study area, notwithstanding, the appearance of AI in Benin in December 2007.

### 3.5. General Discussion

The positive HIT tests on ND in turkeys in this study confirm the clinical presence of this disease in Benin [10]. Turkeys of various ages and sexes from seven villages reacted positively to ND which implies that the wild virus is present in turkey husbandries in the commune of Ouaké. ND is considered the most devastating for village poultry farming [11, 12]. ND remains endemic in Benin and prevails all year round in poultry [6]. Efforts of the veterinary service to have farmers vaccinate poultry are not effective as farmers doubt on the cost effectiveness of the vaccination.

The absence of specific antibodies against sub-types H7N3, H9N2, H5N1, and H5N2 of the AI virus in the HIT reaction excludes presence of this virus in the husbandries sampled for the present study. But epidemiologic monitoring needs to continue since the commune is a border area and is in the proximity of a national park where migratory birds land. 

Controlling the sanitary constraints by vaccinations and other preventive measures [11, 13] will not result in a significant improvement of productivity in village poultry since husbandry practices also constitute a limiting factor [14]. The dramatic reduction of the death rates in one husbandry shows that simple hygienic measures contribute to control of diseases. 

By providing separate housing for the various poultry species, the transfer of infectious agent from less sensitive to more sensitive species can be reduced (ducks are less sensitive to AI than chickens and turkeys). The level of complementary feeding and housing of turkey poults in the majority of the visited husbandries remain insufficient. Scavenging prevails in daytime and farmers do not apply techniques to produce termites or maggots.

 Locally in Burkina Faso [15] and Benin [16], researchers teach small producers to formulate poultry diets by using collected termites or maggots. Feeding the turkey poults a home-made diet makes it possible to maintain them indoors for the first weeks of life; thus, reduce various infections and predation [17]. 

Adjusting the husbandry practices seems simple and not costly but as adoption does not autonomously spread from one farmer to the others, the profitability of disease prevention needs to be checked. Subsequently, the use of appropriate approaches of training and extension on these husbandry practices by the veterinary services and other development organisations might lead to a reduction of turkey mortality and contribute to poverty reduction in this commune. Most extension and training are still top-down paternalistic and use insufficient participatory learning approaches.

## 4. Conclusion

Newcastle disease is a major limiting factor in turkey production in the study area, but avian influenza was not prevalent. Considering the local epidemiologic situation in the area, other studies need to establish an appropriate prevention plan for the dominant pathologies of turkeys. However vaccination alone will not increase productivity of village poultry in view of the multiplicity of related limiting factors such as housing and feeding. 

Improved husbandry practices, together with the distribution of home-made feed, might control pathologies, reduce impact of predation, and increase turkey productivity. The profitability of these actions needs to be demonstrated to the poultry keepers before turkey farming can contribute to poverty reduction.

## Figures and Tables

**Table 1:** Antibody HIT of Newcastle disease in tested blood serum.

Villages	Number tested	Number and % of positives	Titers				
			1 : 16	1 : 32	1 : 64	1 : 128	1 : 256
Badjoudè	20	9 (45)	5	2	1	—	1
Tchaldè	10	8 (80)	1	1	—	3	3
Igbolodè	15	6 (40)	2	3	—	—	1
Awanla	10	4 (40)	1	—	2	1	—
Kpéloudè	15	10 (67)	2	3	2	3	—
Assodè	15	9 (60)	4	2	1	1	1

Totals	85	46 (54)	15	11	6	8	6

**Table 2:** Positive HIT of Newcastle disease according to age and sex in turkeys.

Age (month)	Numbers tested		Positivity (%)	
	Gobbler	Hen	Gobbler	Hen
24	4	5	75	40
17	3	2	67	50
9	4	4	25	25
8	2	3	50	67
7	2	2	50	0

Totals	15	16	—	—

**Table 3:** Result of the HIT on turkey serums for Avian Influenza.

Subtypes of virus	Titres of antigen	Positive/tested (%)
H5N1	1 : 64	0/25 (0%)
H7N3	1 : 128	0/25 (0%)
H9N2	1 : 128	0/25 (0%)
H5N2	1 : 64	0/25 (0%)
